# Ocular Pathology of Oculocerebrorenal Syndrome of Lowe: Novel Mutations and Genotype-Phenotype Analysis

**DOI:** 10.1038/s41598-017-01447-3

**Published:** 2017-05-04

**Authors:** Emilie Song, Na Luo, Jorge A. Alvarado, Maria Lim, Cathleen Walnuss, Daniel Neely, Dan Spandau, Alireza Ghaffarieh, Yang Sun

**Affiliations:** 1 Department of Ophthalmology, Indiana University, Indianapolis, IN USA; 2 Department of Dermatology, Indiana University, Indianapolis, IN USA; 3 Department of Pathology, Indiana University, Indianapolis, IN USA; 4 Department of Ophthalmology, Stanford University, Palo Alto, CA USA; 5 Palo Alto Veterans Administration, Palo Alto, CA USA; 6 Department of Ophthalmology, Stanford University, 1651 Page Mill Road, Rm 2118, Palo Alto, CA 94304 USA

## Abstract

Mutations in the *OCRL1* gene result in the oculocerebrorenal syndrome of Lowe, with symptoms including congenital bilateral cataracts, glaucoma, renal failure, and neurological impairments. *OCRL1* encodes an inositol polyphosphate 5-phosphatase which preferentially dephosphorylates phosphatidylinositide 4,5 bisphosphate (PI(4,5)P_2_). We have identified two novel mutations in two unrelated Lowe syndrome patients with congenital glaucoma. Novel deletion mutations are detected at c.739-742delAAAG in Lowe patient 1 and c.1595-1631del in Lowe patient 2. End stage glaucoma in patient 2 resulted in the enucleation of the eye, which on histology demonstrated corneal keloid, fibrous infiltration of the angle, ectropion uvea, retinal gliosis, and retinal ganglion cell loss. We measured OCRL protein levels in patient keratinocytes and found that Lowe 1 patient cells had significantly reduced OCRL protein as compared to the control keratinocytes. Genotype-phenotype correlation of *OCRL1* mutations associated with congenital glaucoma revealed clustering of missense and deletion mutations in the 5-phosphatase domain and the RhoGAP-like domain. In conclusion, we report novel *OCRL1* mutations in Lowe syndrome patients and the corresponding histopathologic analysis of one patient’s ocular pathology.

## Introduction

The oculocerebrorenal syndrome of Lowe (Lowe syndrome, MIM #309000) is a rare X-linked recessive disease that affects multiple organ systems in male patients^1^. Nearly all patients are afflicted with congenital cataracts, and many have other conditions including glaucoma in 50% of patients and nystagmus in others^2^. Ocular manifestations can include corneal keloids and amblyopia, which severely impair vision and visual development in early childhood^2^. In addition to these ocular findings, Lowe syndrome also causes dysfunction in the kidney and nervous systems^3, 4^. Renal manifestations include low-molecular-weight proteinuria, renal tubular acidosis, hypercalciuria and aminoaciduria, which may ultimately result in renal failure^5, 6^. Neurologic signs consist of severe neonatal hypotonia, mental retardation, and an increased susceptibility to seizures^3, 7–9^. Some patients present with only one of these various phenotypes at birth, which may delay the appropriate diagnosis of the disease^10^. The classic triad of congenital cataracts, infantile hypotonia with intellectual disability, and proximal renal tubular dysfunction often does not present until later in life^11, 12^.

The defective gene *OCRL1* is located on Xq26.1 and encodes an inositol polyphosphate 5-phosphatase; its substrates include phosphatidylinositol-4,5-bisphosphate (PI(4,5)P_2_) and phosphatidylinositol-3,4,5-trisphosphate (PI(3,4,5)P_3_)^13–17^. Most of the disease-causing mutations are located either in the phosphatase domain or the c-terminal RhoGAP domain, which may result in the loss of protein due to the lack of expression or degradation as a consequence of misfolding leading to accumulations of phosphoinositide substrates^7, 18–22^. Currently there have been over 200 mutations that include frameshifts, substitutions, gross inversions, nonsense, and missense mutations, causing a variety of Lowe syndrome phenotypes which range greatly in severity^23–30^. In our previous study, we identified a novel missense mutation (c.1661 A > C; p. D499A) in an 8-year old male patient diagnosed with Lowe syndrome who presented with bilateral congenital glaucoma and cataracts^31^. In this paper, we identified two new deletion mutations in two Lowe syndrome patients with congenital glaucoma and discuss the clinical-pathologic correlation in a patient with end-stage glaucoma.

## Materials and Methods

### Patients and Samples

Two male patients were diagnosed with Lowe syndrome and associated congenital glaucoma at Riley Children’s Hospital, Indianapolis, IN. Patient keratinocyte cell lines were established after informed consent was obtained from the patients’ parents, in accordance with an IRB approved study by Indiana University and is compliant with HIPAA Privacy regulation. The study adhered to the tenets of the Declaration of Helsinki.

### Retinal Imaging

The child’s pupils were dilated with 0.2% cyclopentolate, 0.1% phenylephrine, and 1% cyclopentolate 30 minutes prior to examination under anesthesia. Topical proparacaine (1%) was applied and an eyelid speculum was inserted. Digital images were taken with the RetCam Digital Retinal Camera (Massie Research Laboratories Inc., Pleasanton, CA) using the 130° ROP lens by a study ophthalmologist (DN). All images were stored on the hard-drive of the RetCam machine.

### Keratinocyte isolation

Normal human keratinocytes (NHF588) were isolated from neonatal foreskin tissue as previously described^31^. Isolated keratinocytes were grown in EpiLife Complete media (Cascade Biologics, Portland, OR) supplemented with human keratinocyte growth supplement and 1000 U of penicillin-streptomycin (Roche Molecular Biochemicals, Indianapolis, IN). Lowe patient keratinocytes were cultured under the same conditions.

### Mutation Detection

Patient keratinocyte samples were extracted for RNA and genomic DNA isolation. Total RNA was extracted using the RNAeasy kit. Purified RNA was quantitated with the NanoDrop 2000. Reverse transcription of 2 µg total RNA was performed using SuperScript cDNA synthesis kit with random hexamers. Genomic DNA was isolated and purified using Genomic DNA mini kit. The resulting cDNA and 24 coding exons were amplified and directly sequenced using an ABI 377 DNA sequencer. Mutation assignment was based on the cDNA sequence GenBank NM_000276.3.

### Immunoblot analysis

Keratinocytes lysates were subjected to 4X loading buffer. 40 µg protein were run on 10% gel and transferred to nitrocellulose membrane. Membranes were blocked in 5% non-fat dried milk in PBS. Anti-OCRL and anti-beta-Actin antibodies were used to determine OCRL expression level changes in patient keratinocytes compared to control. IRDye anti-mouse and anti-rabbit (680 and 800) were diluted in concentrations described. An Odyssey Imaging system was used to analyze immunoblots.

### Immunohistochemistry and Immunofluorescence

Enucleated eye sections were stained with Hematoxylin, dehydrated, cleared, and permanently mounted for image acquisition on Nikon ECLIPSE 50i microscope. Immunofluorescence was performed as previously described^31^. Snapshots of histology were taken using a Nikon ECLIPSE 50i microscope equipped with a 20x (numerical aperture 0.4) objective. Images were generated using an attached Rolera Bolt CMOS camera and QCapture Pro 7 software.

### Structure Modeling

UCSF Chimera was used to visualize and construct OCRL protein structures and mutation sites^32^. The PDB accession numbers used were 4CMN (amino acids 215–560), 2QV2 (amino acids 564–901), and 3QBT (amino acids 540–678)^33–35^.

## Results

### Clinical Presentation and Histopathologic analysis

**Figure 1:**
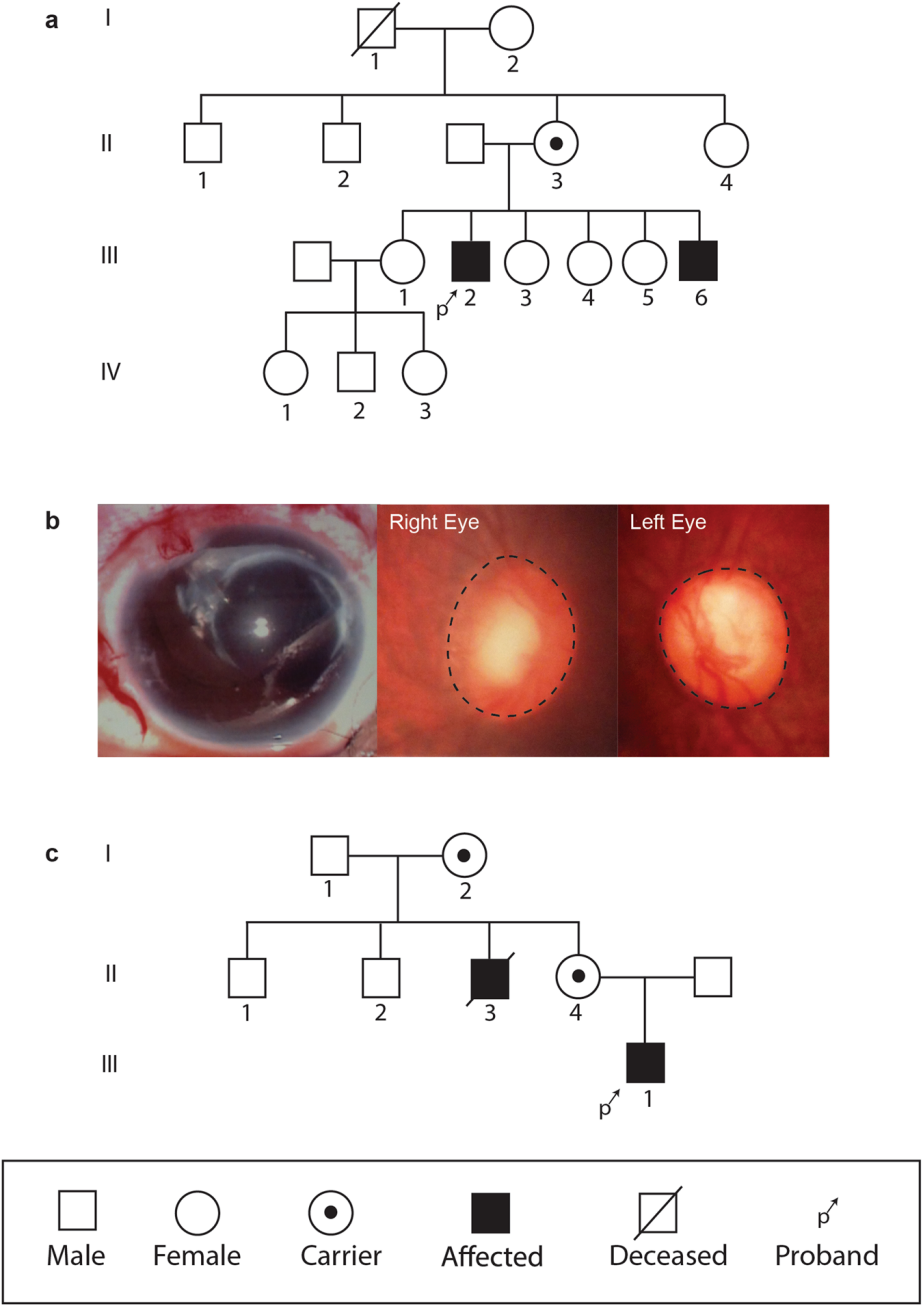
Pedigree and clinical presentation of Lowe patients. (**A**) Pedigree of Lowe patient 1 family, with affected males are denoted by a filled square. Examined female carriers are denoted by dotted circle. (**B**) Slit lamp photo of glaucoma drainage implant in the left eye and optic nerve photos of both eyes in Lowe patient 1. (**C**) Pedigree of Lowe patient 2 family.

Patient 1 (Lowe 1), who has a known history of affected family members (Fig. 1A), presented at 14 days of age with no fix/follow, elevated intraocular pressure (IOP; right eye 31 mmHg, left eye 29 mmHg), corneal edema with Haab striae, and dense bilateral cataracts (Table 1). Corneal diameters were 12 mm and 11.5 mm in the right and left eyes, respectively. Lowe 1 additionally presented with proximal renal tubular acidosis, proteinuria, Dandy Walker Syndrome, congenital muscular dystrophy, developmental delays, low phosphatidylinositol bisphosphate phosphatase (0.3 mmol/min/mg protein [normal range: 2–9]) and keratinocytes with cell growth defects. The patient had multiple eye procedures, including trabeculotomy and lensectomy in both eyes. The left eye required additional surgery – including Ahmed tube shunt implantation, EDTA chelation of calcific band keratopathy, and placement of a Gundersen conjunctival flap – for IOP control and corneal decompensation. Optic nerve photos obtained by pediatric retinal digital camera (RetCam2) showed increased optic nerve cupping with shunt vessel form in the left eye (Fig. 1B). At last follow-up, only the right eye could fix and follow.

**Table 1:** Clinical presentation of Lowe syndrome patients.

	Patient 1		Patient 2	
	*Right Eye*	*Left Eye*	*Right Eye*	*Left Eye*
Visual Acuity at Presentation	Unable	Unable	Blinks to light	Blinks to light
Visual Acuity at Last Visit	Fix/Follow	No Fix/Follow	Fix/Follow	No Fix/Follow
Presenting Intraocular Pressure	31 mmHg	29 mmHg	41 mmHg	44 mmHg
Corneal Diameter (mm)	12	11.5	10.5	10.5
Ocular Findings	Corneal edema with Haab striae		Central nuclear cataract both	
	Dense cataract		Corneal edema	
Systemic Findings	Proximal renal tubular acidosis		Low-set ears	
	Proteinuria			
	Dandy Walker			
	Congenital muscular dystrophy			
	Developmental delay			

Patient 2 (Lowe 2) presented at 2 months of age, with no known affected diagnosis of Lowe syndrome (Fig. 1C). Family history was significant for multiple lenticular flecks in both eyes of his mother and a maternal uncle with congenital cataracts, developmental delay, renal failure, and scoliosis. Vision was wince to light in both eyes but no fix or follow. The patient had elevated IOP (right eye 41 mmHg, left eye 44 mmHg), central nuclear cataracts in both eyes, and corneal edema. Corneal diameter was 10.5 mm in both eyes (Table 1). The patient was also noted to have low-set ears. The patient underwent cataract extraction in both eyes, anterior vitrectomy and pupilloplasty in the right eye, and endocyclophotocoagulation and trabeculotomy in the left eye. After trabeculotomy, the left eye developed a large hyphema and retinal detachment which required anterior chamber washout and penetrating keratoplasty. The left eye subsequently underwent placement of an Ahmed glaucoma drainage device; however due to progressive decreasing vision to no light perception and pain, the eye was enucleated for pain relief.

**Figure 2:**
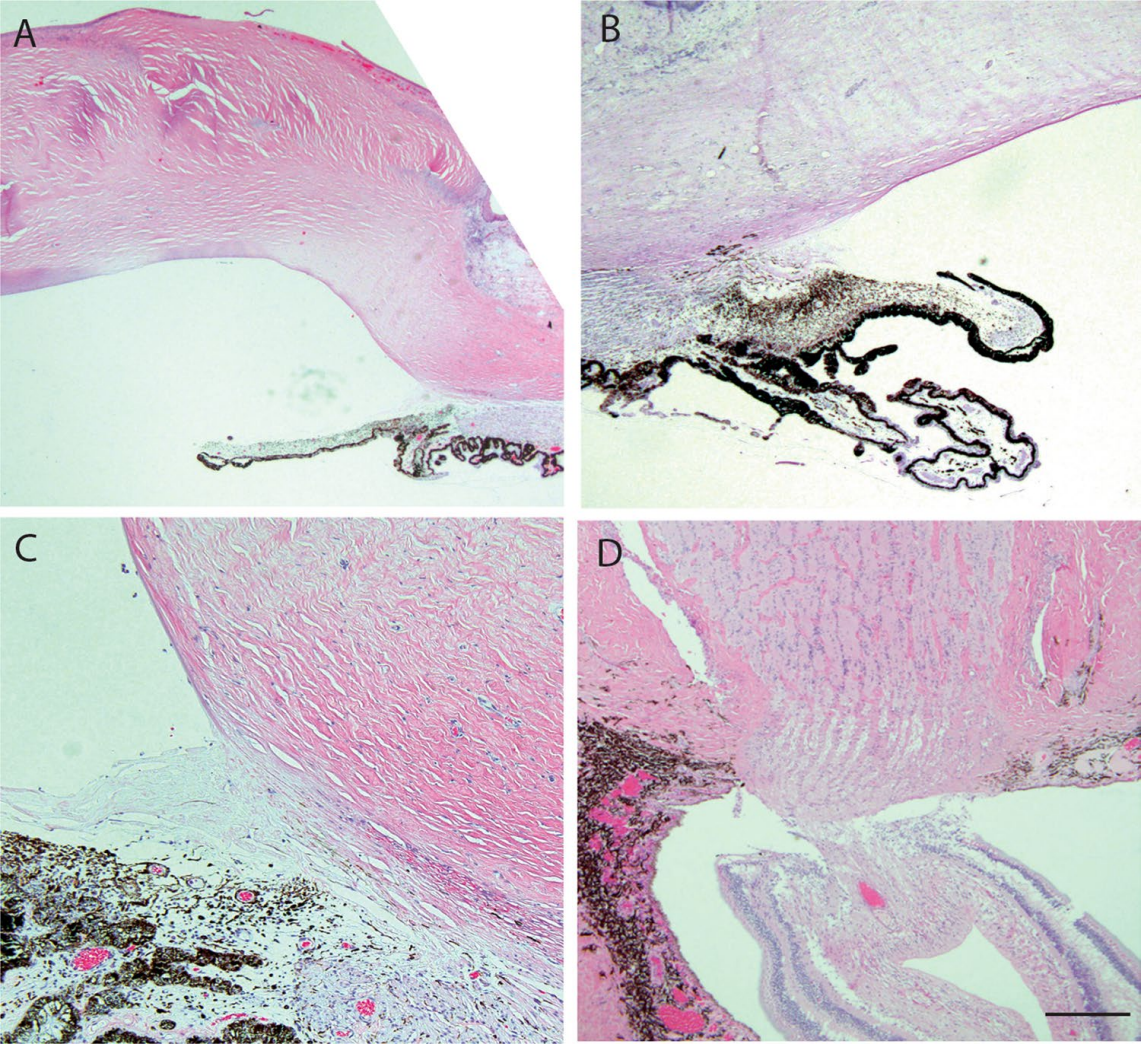
Ocular histopathology analysis of Lowe patient 2. (**A**) Cornea thickening and scarring with fibrovascular proliferation in the subepithelial region. Scale bar 1 mm. (**B**) The angle of the anterior chamber is covered in dense fibrous tissues adherent to the cornea and the iris stroma. Extensive ectropion uvea overlay the atropic iris. Scale bar 1 mm. (**C**) Ciliary body is underdeveloped and atrophic with peripheral staphyloma. Scale bar 0.5 mm. (**D**) Optic nerve shows loss of retinal ganglion cell layer, retinal gliosis, and cupping of optic nerve head. Scale bar 0.5 mm.

**Figure 3:**
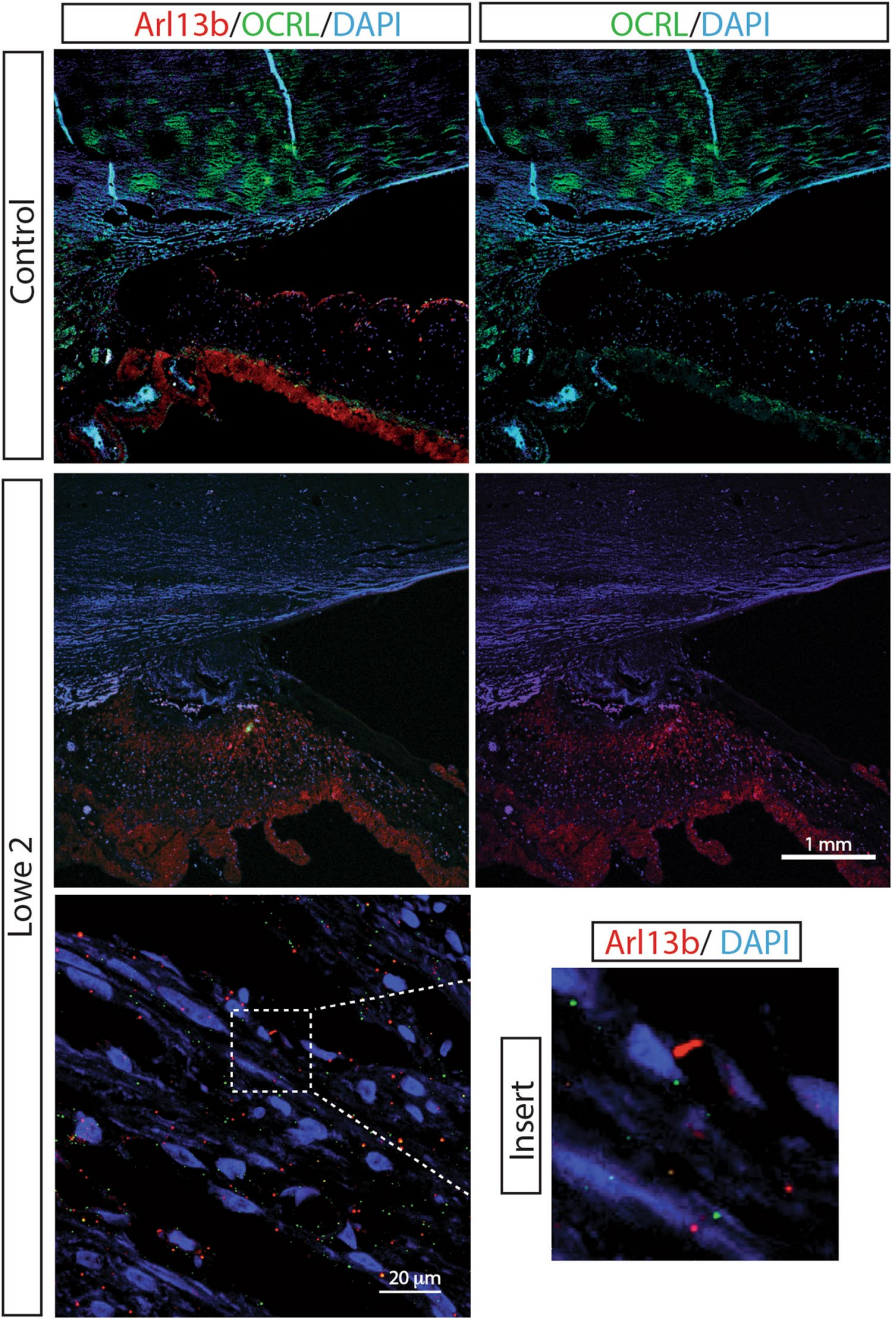
Absent OCRL staining in ocular tissue of Lowe patient 2. Immunofluorescence analysis of the trabecular meshwork in normal human eye (control) and the eye from Lowe patient 2, both stained for Arl13b (red) and OCRL (green), DAPI staining in blue. Scale bar 1 mm.

On histopathology of the enucleated eye, the cornea is completely opacified with extensive fibrovascular proliferation, loss of endothelial cells, and peripheral stromal neovascularization, indicating corneal ischemia (Fig. 2A). The angle of the anterior chamber is covered with dense fibrous tissue with peripheral anterior synchiae. The iris stroma is atrophic with extensive ectropion uvea (Fig. 2B,C). Ciliary body is atrophic with peripheral staphyloma under the area of Ahmed glaucoma drainage implant. Optic nerve shows atrophy and loss of axons and cupping of optic nerve head (Fig. 2D). Upon immunofluorescence staining with OCRL specific antibodies and anti-Arl13b (a small GTPase) antibodies, a marked decrease in OCRL signal was noted in the trabecular meshwork of the Lowe syndrome patient tissue, as compared to the normal human cadaveric control eye (Fig. 3).

### Mutations Analysis in Lowe patients

**Figure 4:**
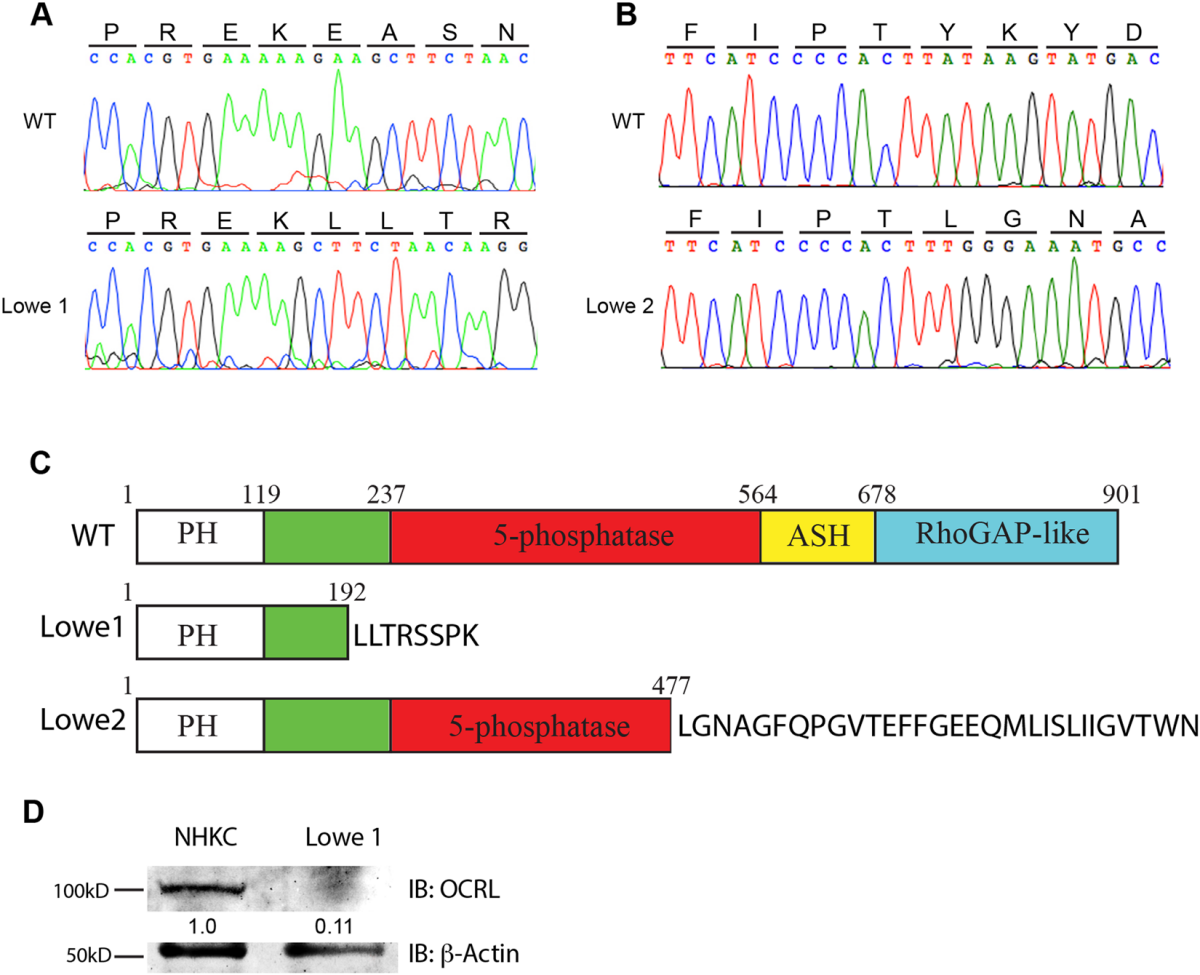
Mutation identification of two Lowe syndrome patients with congenital glaucoma. (**A**) DNA sequencing of the *OCRL1* gene in keratinocytes from Lowe patient 1 revealed a novel 4-basepair deletion mutation of gene OCRL (c.739-742delAAAG, p. Lys192Lys fsX8) in exon 8. (**B**) DNA sequencing of the OCRL gene in keratinocytes from Lowe patient 2 revealed a novel 37-basepair deletion mutation at the tail of exon 14 of OCRL (c. 1595-1631del, p. Tyr477Leu fsX). (**C**) The 4-basepair deletion at exon 8 in Lowe patient 1 caused a reading frame shift with 8 unpredicted amino acids at amino acid 192 in the region between PH and the 5-phosphatase domain resulting in size change from 901 to 200 amino acids. In patient 2, the 37-baspair deletion at the tail of exon 14 caused a reading frame shift at amino acid 477 in the 5-phosphatase domain, resulting in a protein length change to 506 amino acids. (**D**) OCRL protein expression levels were measured using Western Blots of patient and normal control keratinocyte extracts. Lowe 1 had a significantly reduced OCRL protein content compared to the control.

DNA sequencing of gene *OCRL1* revealed a novel deletion mutation in Lowe 1 (c.739-742delAAAG, p.Lys192Lys fsX)(Fig. 4). This 4-basepair deletion site localized in exon 8, which caused a shift of reading frame after Lys at #192 amino acid in the region between PH and 5-phosphatase domain. This deletion mutation resulted in a translational size change for OCRL protein from 901 to 200 amino acids.

We also identified another novel deletion mutation in Lowe 2 patient (c. 1595-1631del, p. Tyr477Leu fsX) (Fig. 4B). This 37-basepair mutation site localized at the tail of exon 14, which caused another reading frameshift at amino acid 477 in the 5-phosphatase domain, resulted in a truncated protein of 506 amino acids.

### OCRL Protein Analysis for Lowe patients

OCRL is a multi-domain protein comprising an N-terminal pleckstrin homology (PH) domain followed by a central inositol-5-phosphatase domain, an ASPM-SPD-2-Hydin (ASH) domain, and a C-terminal, catalytically inactive RhoGAP-like domain. To test OCRL protein function in the patients’ cells, we extracted Lowe patient and normal control keratinocytes and performed immunoblot analysis. The amount of OCRL was estimated in total cell extracts using the total amount of protein for normalization. As shown in Fig. 4D, Lowe 1 patient with 4-basepair deletion mutation in the *OCRL1* gene had a markedly reduced content of OCRL when compared to control. Thus the mutation in OCRL in Lowe patient 1 resulted in unstable protein and decreased wildtype expression.

### Genotype-phenotype analysis for glaucoma in Lowe syndrome patients

**Figure 5:**
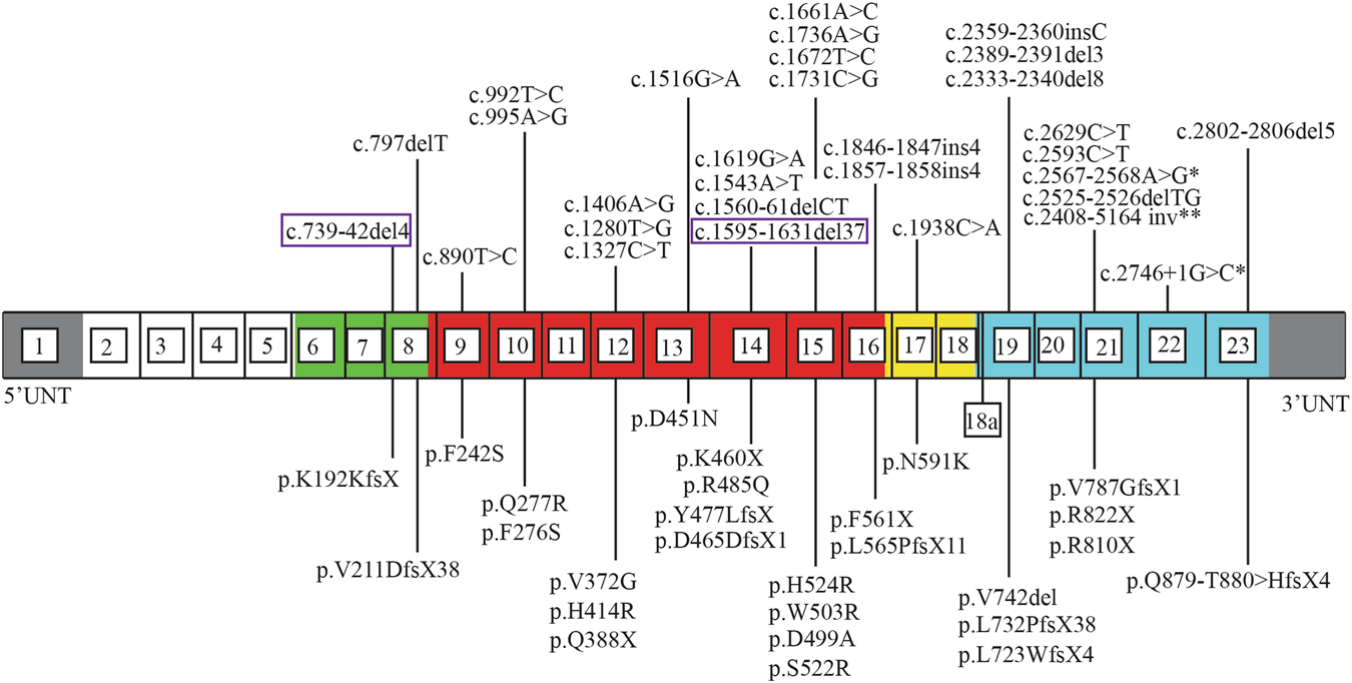
Mapping of known mutations associated with congenital glaucoma in *OCRL1* gene and OCRL protein domains. Thirty *OCRL1* mutations, including the two we identified in this paper, have been identified so far in Lowe Syndrome patients with congenital glaucoma. These mutations are the result of a variety of mutations including insertion, deletion, and inversion mutations which have caused a spectrum of reading frame shifts, truncated proteins, and disease phenotypes. The two novel mutations discussed in this paper are highlighted by the purple rectangles. *Refers to intronic mutations that caused splicing defects. **Refers to a mutation that caused gross inversion. Exons including the alternatively spliced exon 18a are numbered 1 to 23. The PH domain is shown in white, 5-phophatase domain is indicated in red, the ASPM-SPD-2Hydin (ASH) domain in yellow, the RhoGAP-like domain in blue, the linkage domain between the PH and 5-phophatase domain in green, and the untranslated regions in yellow.

To date, 30 *OCRL1* mutations have been identified in Lowe Syndrome patients with congenital glaucoma, including the two patients identified in this paper (Table 2). These 30 mutations exhibit a variety of changes that include not only missense and nonsense, but also insertion, deletion, and gross inversion mutations which cause shifts of reading frame and result in truncated proteins. Half of these (16 mutations) occur in exons from 9 to 16, which encode the 5-phosphatase domain of OCRL protein, with most of them (11 mutations) are single base pair missense change. One third of identified (10 mutations) occur in exons from 18 to 23, which encode RhoGAP-like domain in C terminal, and most of these mutations cause truncated OCRL protein. Only two mutations occur in exons 16 and 17 coding ASH domain, and two deletion mutations occur in the 8^th^ exon coding the linkage domain between PH and 5-phosphatase domains (Fig. 5).

**Table 2:** OCRL mutations associated with congenital glaucoma.

Patient Number	Mutation Type	Exon	Nucleotide Change	Protein Change	Literature
1	Deletion	8	c.739-742delAAAG c.741-744delAGAA	p.Lys192Lys fsX	Lowe I
2	Deletion	14	c.1595-1631del37	p.Tyr477Leu fsX	Lowe II
3	Missense	15	c.1661A > C GAC > GCC	p.Asp499Ala	Luo 2014
4	Missense	15	c.1736A > G CAC»CGC	p.His524Arg	Draaken 2011
5	Deletion	8	c.797delT GTA»GAC	p.Val211Asp fsX	Hichri 2010
6	Missense	9	c.890 T > C TTT»TCT	p.Phe242Ser	”
7	Missense	12	c.1280 T > G GTC > GGC	p.Val372Gly	”
8	Missense	12	c.1406 A > G CAT > CGT	p.His414Arg	”
9	Missense	13	c.1516 G > A GAC > AAC	p.Asp451Asn	”
10	Nonsense	14	c.1543 A > T AAA > TAA	p.Lys460X	”
11	Deletion	14	c.1560-1561delCT GACTTC > GATC	p.Asp465Asp fsX	”
12	Missense	15	c.1672T > C TGG > CGG	p.Trp503Arg	”
13	Insertion	16	c.1846-1847insGACT TTC > TGACTTC	p.Phe561X	”
14	Insertion	16	c.1857-1858insCCTT TCCTTA > TCCCCTTTTA	p.Leu565Pro fsX	”
15	Missense	17	c.1938C > A AAC > AAA	p.Asn591Lys	”
16	Insertion	19	c.2359-2360insC CTT > CCT	p.Leu732P fsX	”
17	Deletion	19	c.2389-2391delGTA	p.Val742del	”
18	Nonsense	21	c.2629 C > T CGA > TGA	p.Arg822X	”
19	Missense	22	c.2746 + 1 G > C	Splicing defect	”
20	Deletion	23	c.2802-2806delGACTC CAGACTCCA > CACA	p.Gln879-Thr880 > His fsX	”
21	Missense	10	c.995 A > G CAA > CGA	p.Gln277Arg	Sethi 2008
22	Deletion	21	c.2525-2526delTG GTGGCT > GGCT	p.Val787Gly fsX	”
23	Missense	21	c.2567-2568 A > G	Splicing defect	”
24	Deletion	19	c.2333-2340del8 TGGATGAA TTGGATGAAGG > TGG	p.Leu723Trp fsX	Kim 2014
25	Missense	14	c.1619G > A CGG > CAG	p.Arg485Gln	”
26	Nonsense	21	c.2593 C > T CGA > TGA	p.Arg810X	”
27	Inversion	21	c. 2408-5164 inv	Gross inversion	”
28	Nonsense	12	c.1327 C > T CAA > TAA	p.Gln388X	Kubota 1998
29	Missense	15	c.1731C > G AGC > AGG	p.Ser522Arg	”
30	Missense	10	c.992 T > C TTC > TCC	p.Phe276Ser	Chabaâ 2006

**Figure 6:**
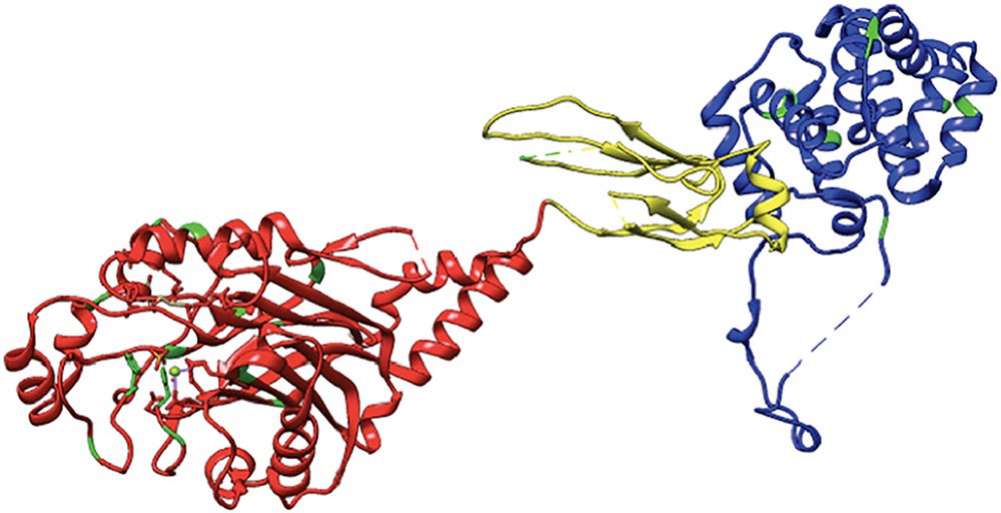
Structural analysis of mutations associated with congenital glaucoma in OCRL. Mutations in OCRL 5-phosphatase domain (red), ASH domain (yellow) and Rho-GAP like domain (blue) are mapped in the crystal structure of OCRL, showing majority of mutations (green) within the 5-phosphatase domain. The PDB accession numbers used were 4CMN (amino acids 215–560), 2QV2 (amino acids 564–901), and 3QBT (amino acids 540–678).

Three-dimensional structural analysis of the OCRL shows that only 4 out of the 16 mutations in the 5-phosphatase domain are in or near the active site, while only 2 of the amino acid mutation sites directly localize to the active site (Fig. 6). This suggest while the loss of 5-phosphatase activity alone not required for the glaucoma phenotype.

## Discussion

While ocular phenotype of Lowe syndrome has been recognized since the initial description of the disease^1^, little is known of the ocular pathophysiology of cataract, glaucoma, and corneal keloid formation. We here report the identification of two novel mutations of *OCRL1* in affected Lowe syndrome patients, present the corresponding histopathologic analysis of an enucleated eye, and perform a genotype-phenotype analysis of mutations resulting in congenital glaucoma in Lowe syndrome.

We identified two new deletion mutations, c.739-742delAAAG and c. 1595-1631del, in the *OCRL1* gene in two unrelated patients with Lowe Syndrome. Patient 1’s deletion occurs in exon 8, just preceding the 5-phosphatase domain, which results in unstable transcribed protein and the loss of OCRL activity as demonstrated by PI(4,5)P2 activity. Patient 2’s deletion occurs within exon 14, directly affecting the 5-phosphatase domain, which would lead to incomplete transcription of the ASPM-SPD-2-Hydin (ASH) domain, and the C-terminal, catalytically inactive RhoGAP-like domain. Lowe 1 had a four base pair deletion mutation in the region between the PH and 5-phosphatase domains, causing the OCRL protein to shorten from 901 amino acids to 200 amino acids and lose its inositol 5-phosphatase domain. This frameshift mutation also caused Lowe 1 to have significantly reduced levels of OCRL in his keratinocytes as compared to controls. The deletion mutation in Lowe 2 occurred in the 5-phosphatase domain and caused a frameshift that shortened the OCRL protein to 506 amino acids. Both patients presented with bilateral congenital cataracts and elevated intraocular pressure that required a number of glaucoma surgeries.

It is recognized that the majority of *OCRL1* mutations that cause Lowe syndrome are deletion and early truncation mutations, and a smaller number of mutations are missense mutations that may shed more light on the functional importance of each of the domains^36^. Most of the currently reported mutations for Lowe syndrome (eye, brain, and kidney) are located within the 5-phosphatase domain and the RhoGAP-like domain, whereas the mutations associated with Dent’s disease (kidney alone) are more in the N-terminal domain preceding the catalytic and C-terminal lipid binding domains^36^. Based on our analysis of the glaucoma associated mutations, the majority of missense mutations occur within the 5-phosphatase domain, and only 30% are found within the RhoGAP-like domain, suggesting that the enzymatic functions of 5-phosphatase are important in causing the glaucoma phenotype. However, on further structural analysis based on the location of mutated amino acids, only 4 out of 16 missense mutations were in the active site or near the core of the catalytic domain of 5-phosphatase. Further, the symptoms of the patients were not more severe than that of mutations occurring outside of the 5-phosphatase domain. This raises an important question that has been noted by several groups, namely, how important is the 5-phosphatase function in the cause of Lowe syndrome, if PI(4,5)P2 levels are high also in the Dent’s disease patient derived fibroblasts.

One of the two patients we report here does not manifest any renal phenotype. In fact, the most severe organ system involved is the eye, with advanced glaucoma leading to complete blindness and requiring removal of the eye. The pathology studies support the findings of previous Lowe syndrome eyes at autopsy, namely the corneal keloid formation, iris anomalies of ectropion uvea, ciliary body cysts, and retinal gliosis near the optic nerve^37–40^. The advances in cataract surgery have led to the successful removal of the lens, which in previous reports have found retained nuclei, thickened lens capsule, formation of excrescences under the lens epithelial cells^37, 41, 42^. This deletion mutation presented with severe ocular disease but no significant renal pathology, raising the possibility of disease-modifying genes that could be expressed in the kidney and ameliorate the loss of OCRL.

In conclusion, we here report two novel mutations in *OCRL1* with severe ocular phenotypes of congenital glaucoma, with one patient requiring enucleation. Analysis of the mutations that are associated with glaucoma in Lowe syndrome showed predominant distribution of mutations within the 5-phosphatase and the RhoGAP-like domain.
